# Exploration of editorial board composition, Citescore and percentiles of Hindawi journals indexed in Scopus

**DOI:** 10.1016/j.dib.2018.05.066

**Published:** 2018-05-19

**Authors:** Hilary I. Okagbue, Aderemi A. Atayero, Muminu O. Adamu, Sheila A. Bishop, Pelumi E. Oguntunde, Abiodun A. Opanuga

**Affiliations:** a Department of Mathematics, Covenant University, Canaanland, Ota, Nigeria; b Department of Electrical and Information Engineering, Covenant University, Canaanland, Ota, Nigeria; c Department of Mathematics, University of Lagos, Akoka, Lagos, Nigeria

**Keywords:** Hindawi, Bibliometrics, Data analysis, Scopus, Percentile, Smart campus, Ranking analytics, Statistics, Citescore

## Abstract

The statistical analysis of editorial board composition, Citescore and percentile of 180 Hindawi journals currently indexed in Scopus are presented in this data article. The three indicators (editorial board composition, Citescore and percentile) can be helpful for researchers to make informed decision about the impact of Hindawi journals. The last two indicators are components of Scopus Citescore metrics.

**Specifications Table**

**Table:** 

Subject area	Decision Sciences
More specific subject area	Bibliometrics, Statistical data analysis
Type of data	Table, Figure and MS Excel
How data was acquired	The data was obtained from freely open access hindawi journals
Data format	Raw, partially analyzed
Experimental factors	Patterns of distribution of editorial board members, Citescore and percentiles of journals indexed in Scopus.
Experimental features	Only the Journals indexed in Scopus were considered
Data source location	Hindawi Publisher
Data accessibility	All the data are in this data article

**Value of the data**

• The data could be in helpful in monitoring imbalances in editorial board composition across the continents.
• The data could be helpful in monitoring the performances of journals over time.
• The data could be helpful in making informed decisions by researchers.
• The data can be used in bibliometric analysis.

## 1 Data

**Fig. 1:**
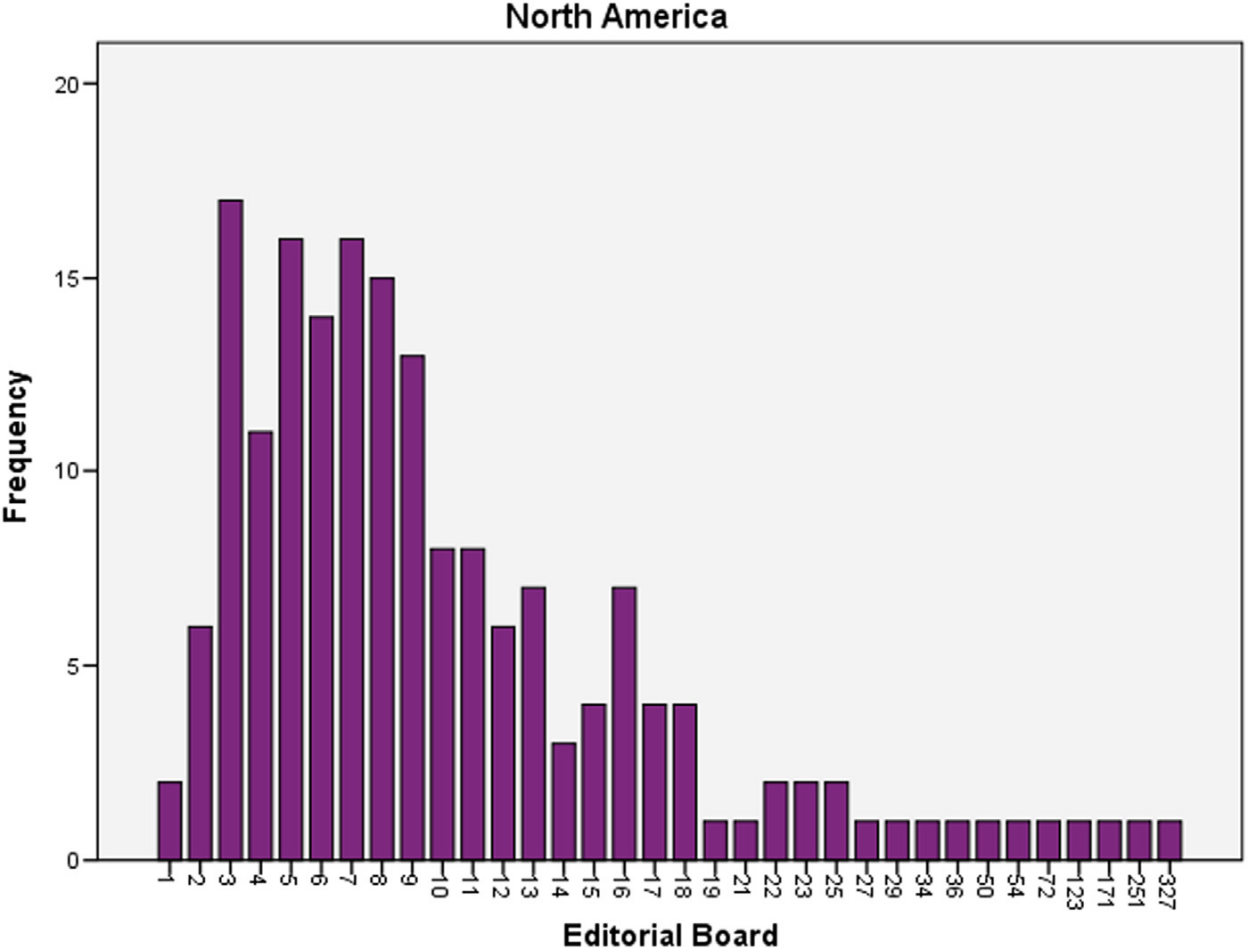
Editorial Board members with affiliations in North America.

**Fig. 2:**
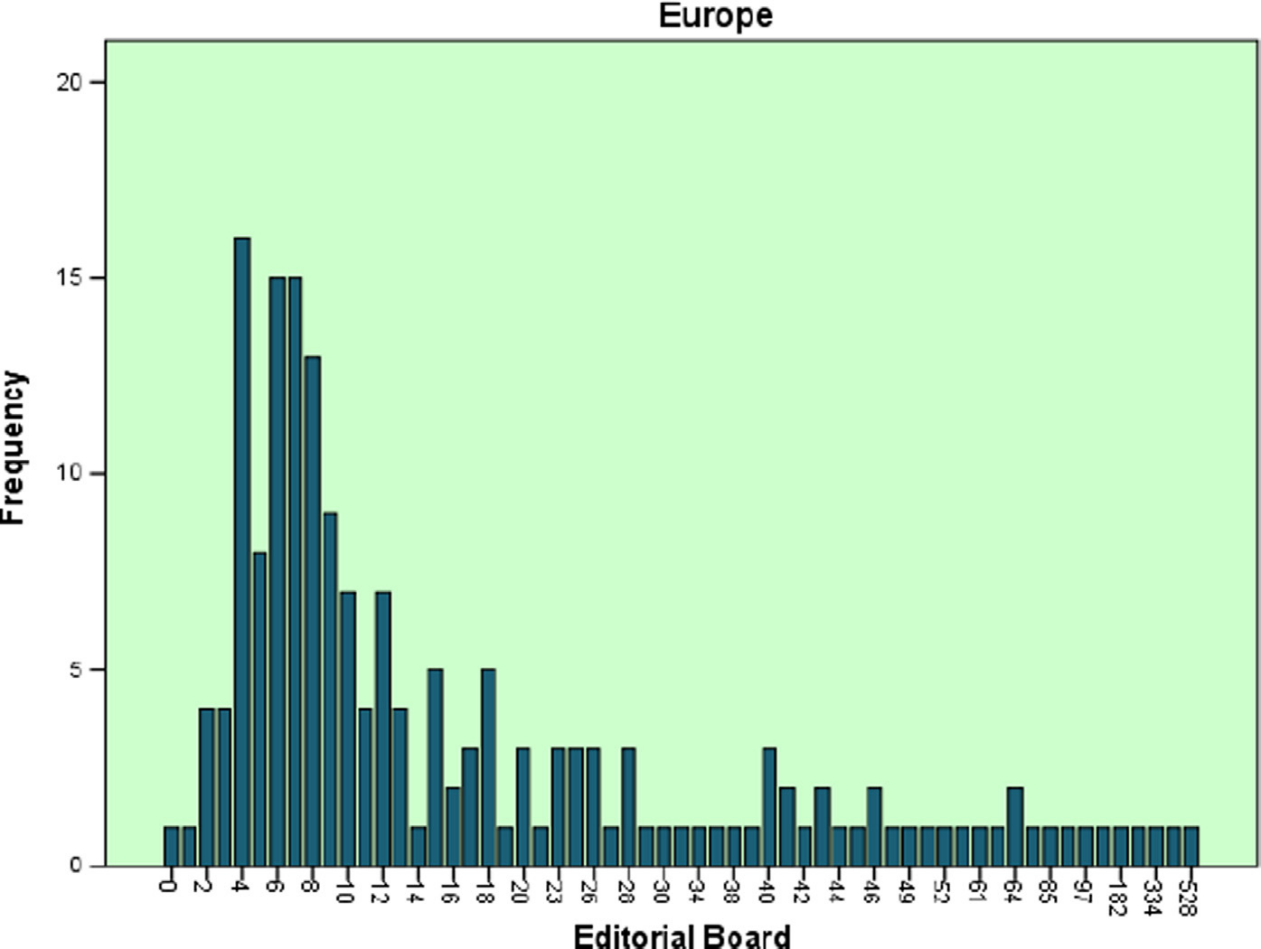
Editorial Board members with affiliations in Europe.

**Fig. 3:**
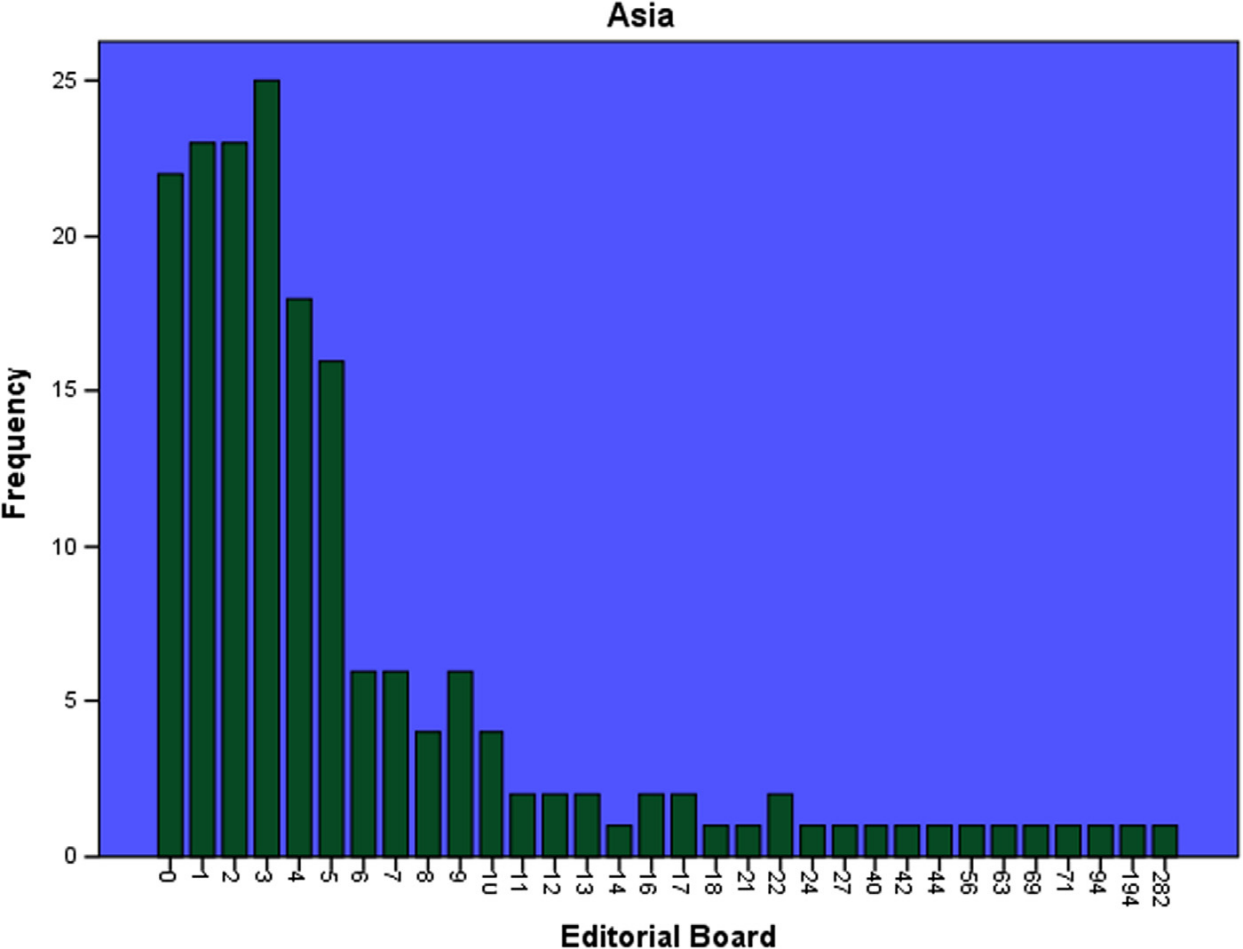
Editorial Board members with affiliations in Asia.

**Fig. 4:**
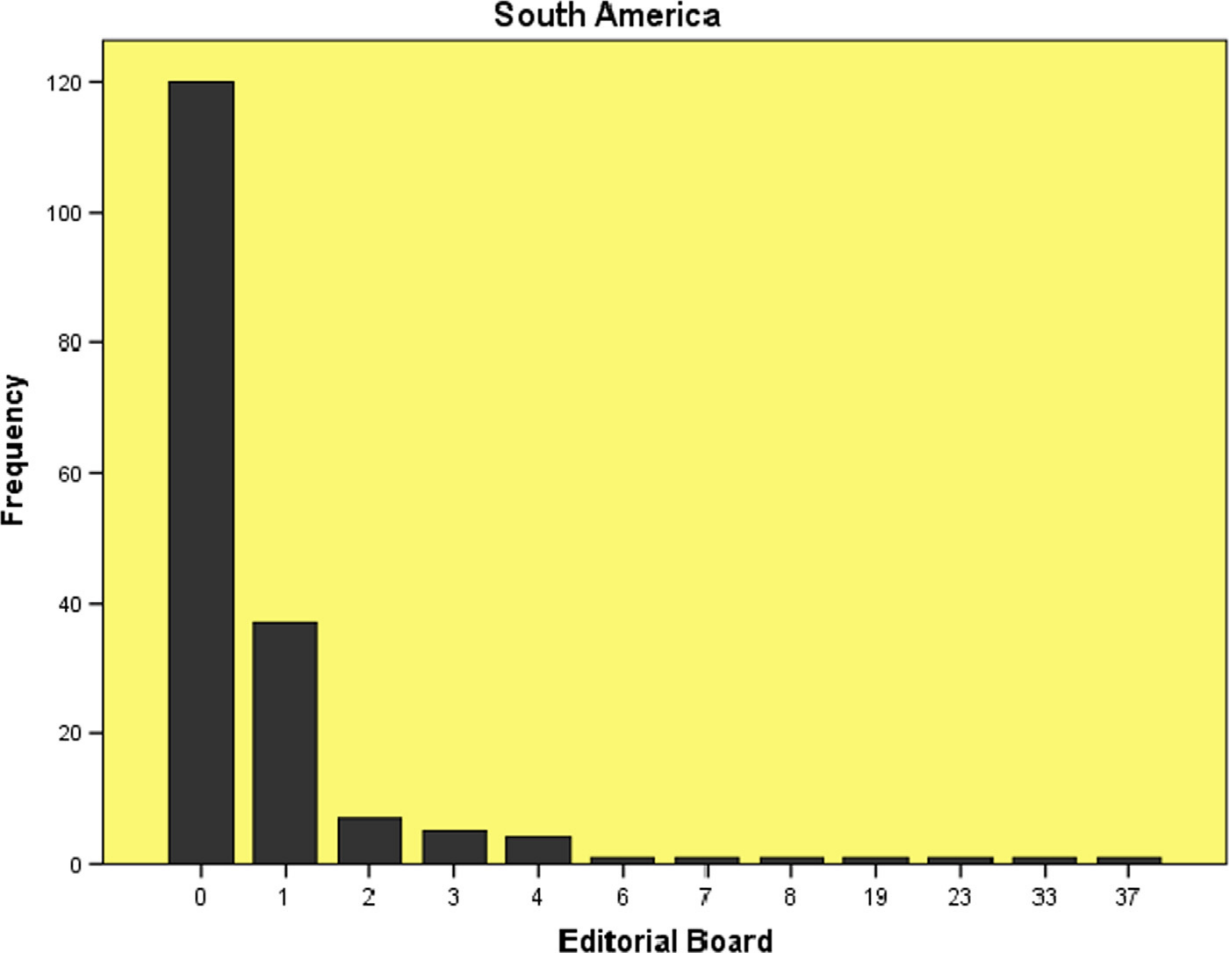
Editorial Board members with affiliations in South America.

**Fig. 5:**
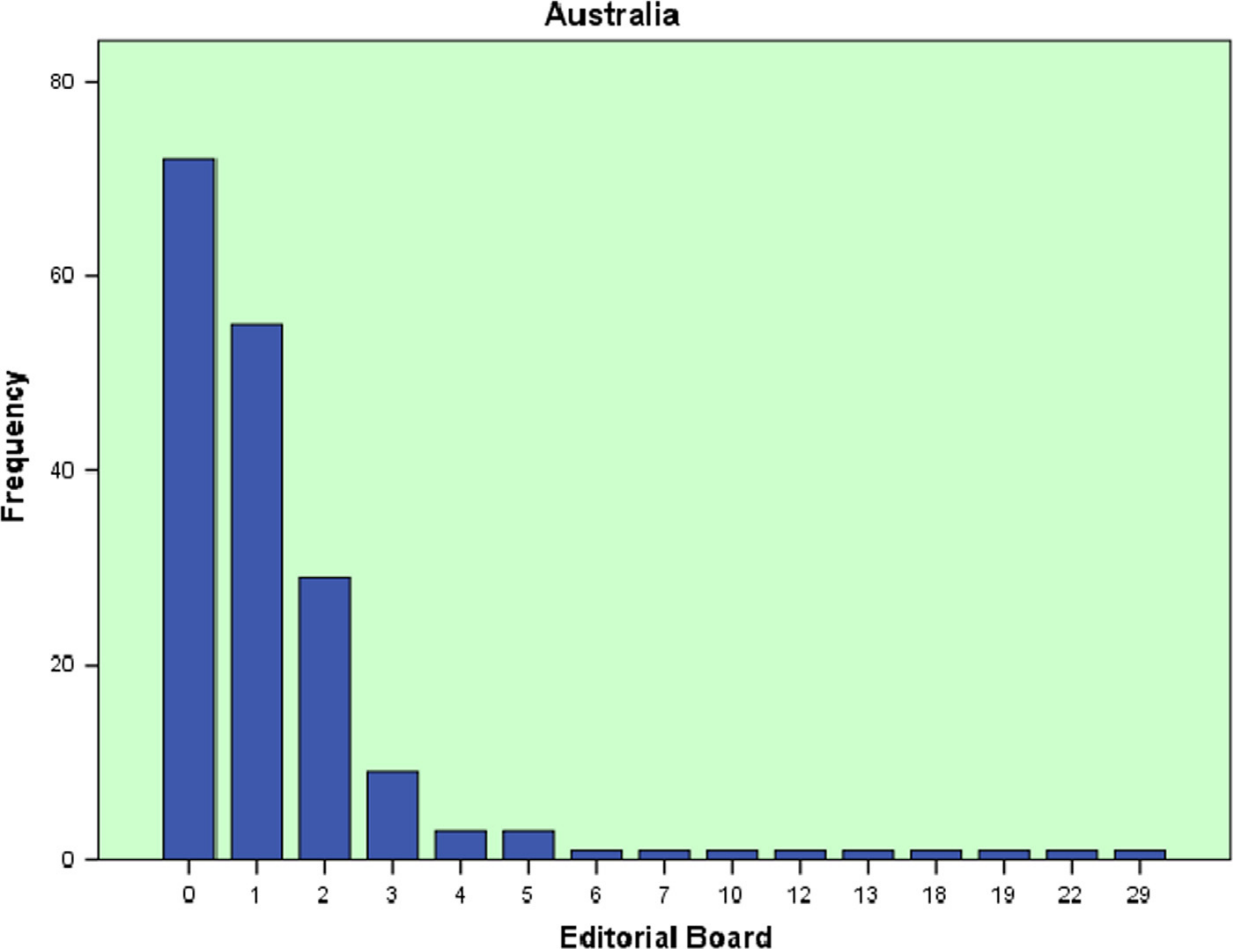
Editorial Board members with affiliations in Australia.

**Fig. 6:**
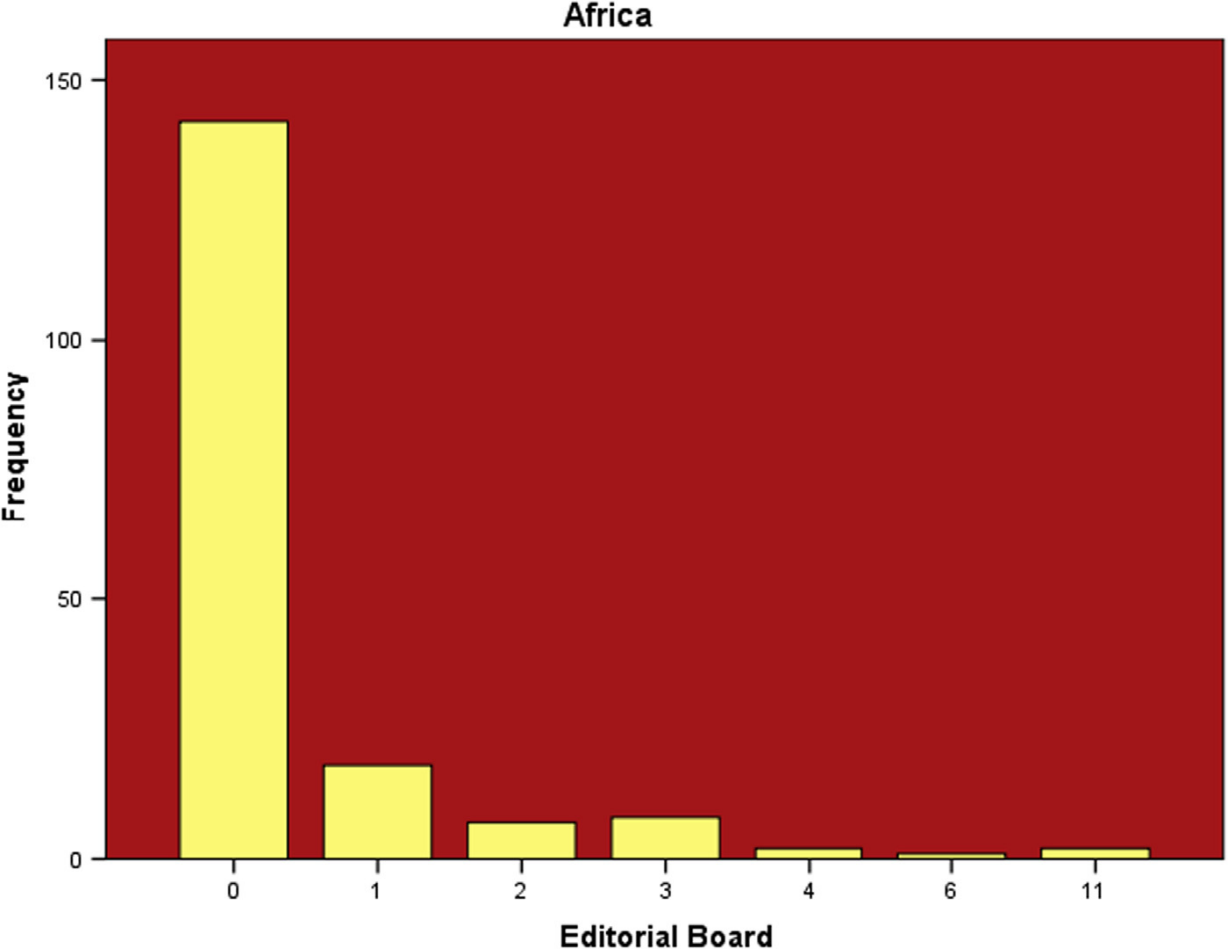
Editorial Board members with affiliations in Africa.
c. The summary statistics of the total number of editorial board members presented in Fig. 7.

The datasets contained in this article are listed as follows:

a. The dataset of editorial distribution of 180 Hindawi journals indexed in Scopus. This can be assessed as Supplementary data.
b. The frequency of editorial board composition of the 180 Hindawi journals and their summary statistics (Table 1). These are also presented as bar charts (Fig. 1, Fig. 2, Fig. 3, Fig. 4, Fig. 5, Fig. 6).

**Table 1:** Summary statistics of editorial composition of Hindawi journals indexed in Scopus.

	North America	Europe	Asia	South America	Australia	Africa
N	180	180	180	180	180	180
Mean	14.78	27.36	9.67	1.19	1.68	0.51
Std. Error of Mean	2.536	4.534	2.079	0.326	0.271	0.109
Median Q2	8	10	3	0	1	0
Mode	3	4	3	0	0	0
Std. Deviation	34.028	60.83	27.89	4.367	3.641	1.459
Variance	1157.903	3700.309	777.853	19.074	13.257	2.128
Skewness	7.013	5.826	7.242	6.418	4.853	4.89
Kurtosis	54.534	38.728	61.381	44.554	27.281	29.805
Range	326	528	282	37	29	11
Minimum	1	0	0	0	0	0
Maximum	327	528	282	37	29	11
Sum	2661	4924	1741	215	303	92
Percentiles Q1	5	6	1.25	0	0	0
Q3	13	25.75	7	1	2	0

**Fig. 7:**
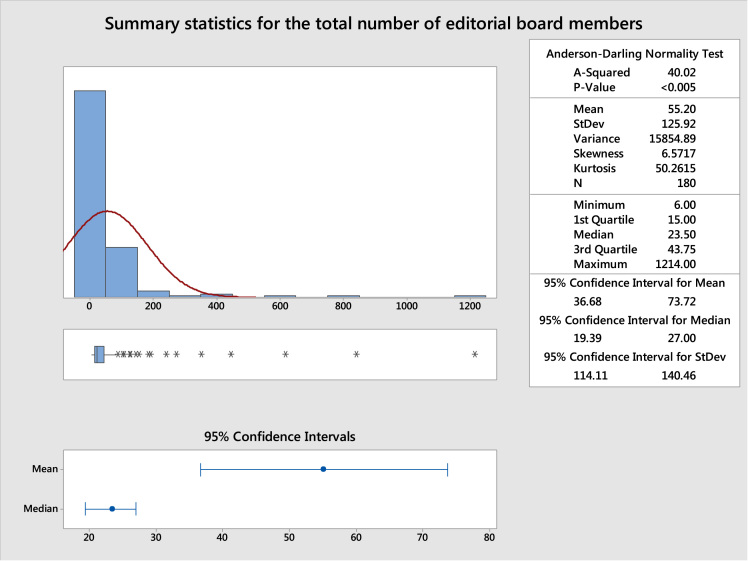
Summary statistics of the total number of editorial board members for the 180 journals.
d. The summary statistics of the Citescore values of the 180 Hindawi journals shown in Fig. 8.

**Fig. 8:**
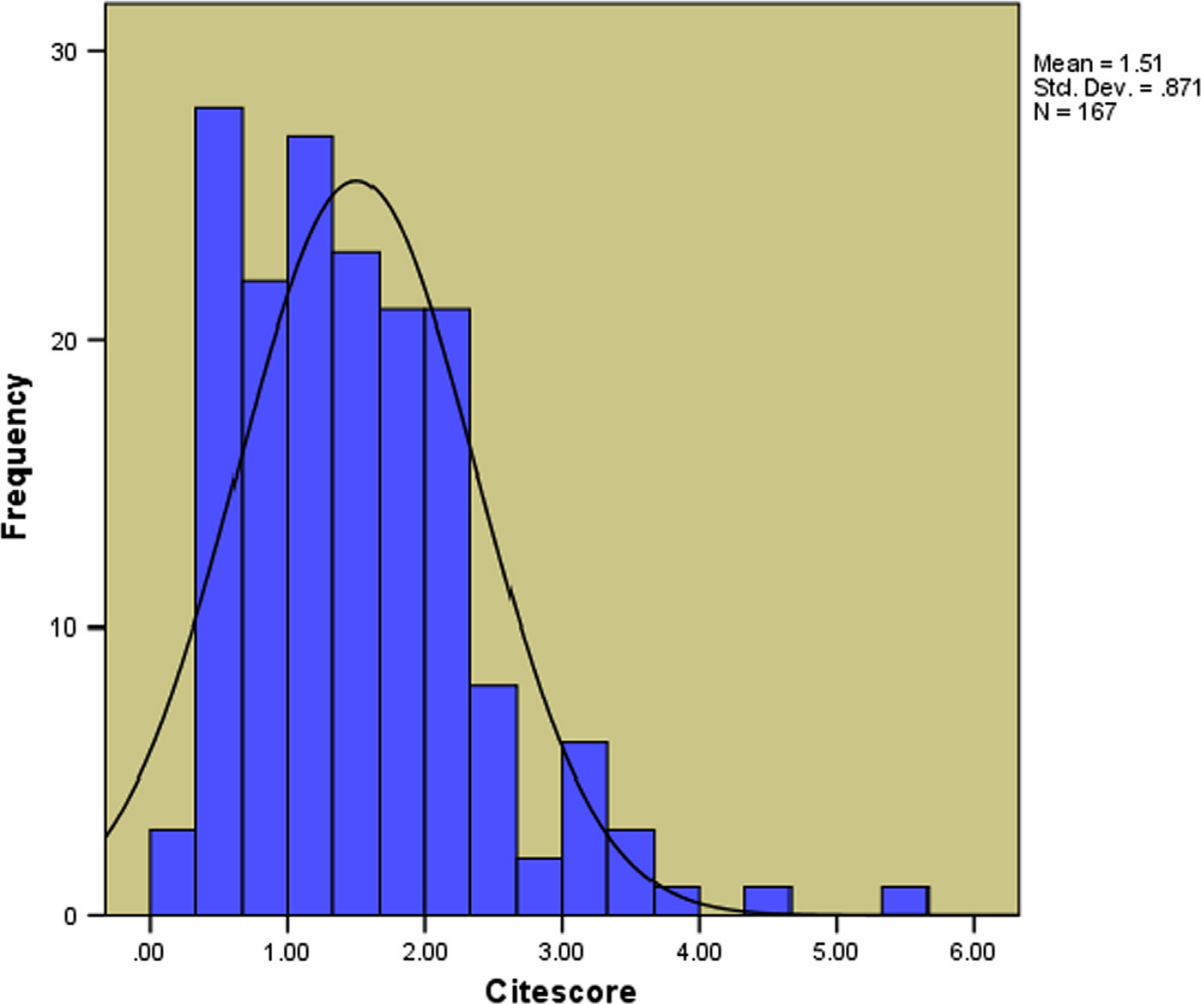
The distribution of the Citescore. Remarks: The average, median, standard deviation, skewness and kurtosis of the Citescore for the 180 Hindawi journals indexed in Scopus are computed to be 1.506287, 1.38, 0.870669, 1.234816 and 2.624597 respectively. Also, 13 journals are yet to be given their Citescore by Scopus.
e. The summary statistics of the percentile values of the 180 Hindawi journals shown in Fig. 9.

**Fig. 9:**
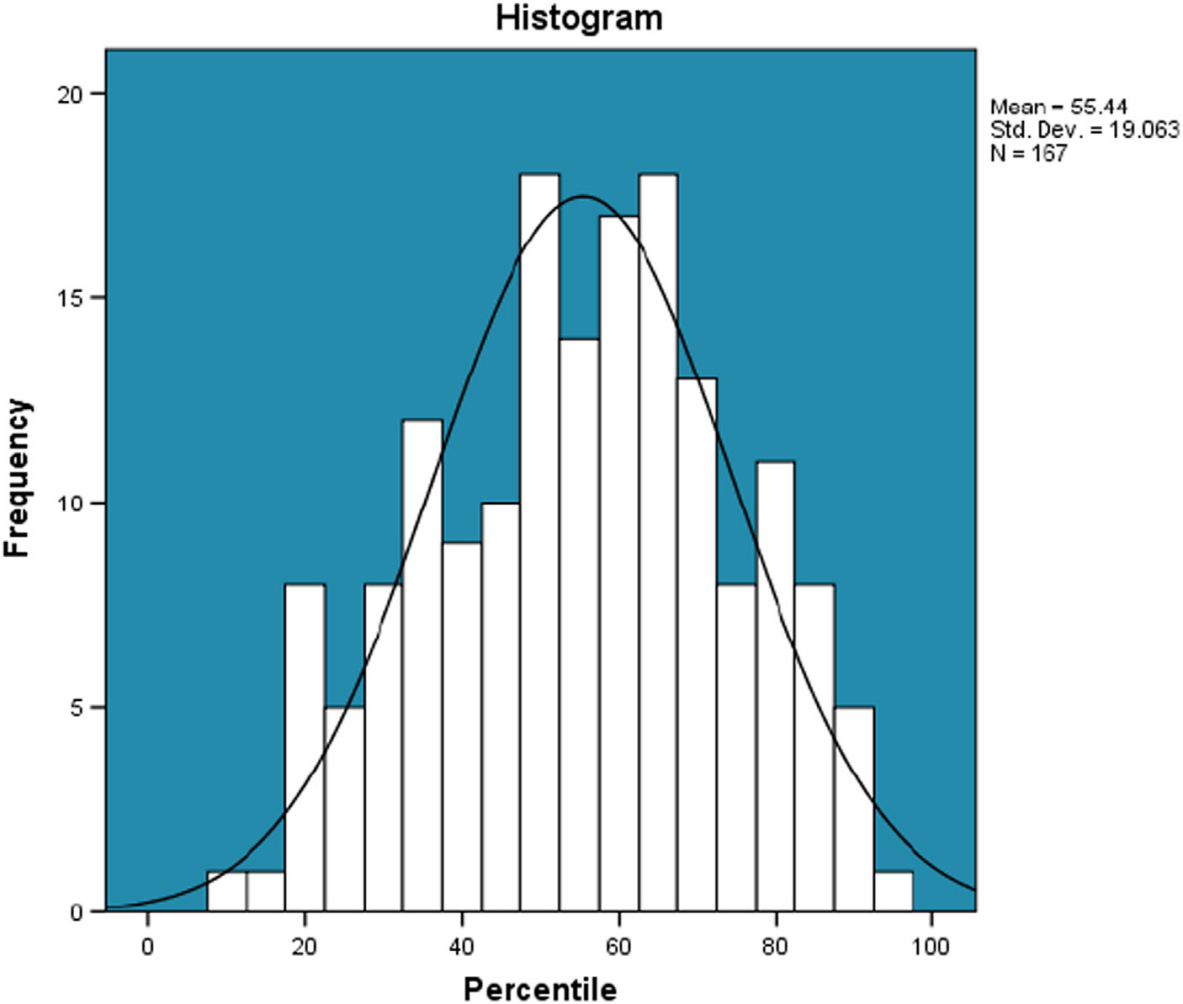
The distribution of the Percentile. Remarks: The average, median, standard deviation, skewness and kurtosis of the percentile for the 180 Hindawi journals indexed in Scopus are computed to be 55, 57, 19, −0.163294 and −0.620154 respectively. Also, 13 journals are yet to be given their percentile by Scopus.

### 1.1 Detailed data description

Currently, Hindawi publishing Corporation publishes 180 journals indexed in Scopus. Scopus is a citation and abstract database launched in 2004 but covers records of previous years dating as far as 1950. The database is provided and managed by Elsevier and currently holds over 70 million records of peer reviewed articles, reviews, notes, editorials, survey, book and book chapters, monographs, patents and conference proceedings of publishers of all academic domains. Scopus uses four quality assessment measures to rank and determine the impact of journals indexed in it. These include: h-index, Citescore, SJR (SCImago Journal Rank) and SNIP (Source Normalized Impact per Paper). A database called SciVal uses data mining to analyze the indexations in Scopus.

Citescore is a subset of Citescore metrics launched as a new metric to track the performance of journals over time while the journal percentile maps the Citescore into a 100 percentage scale which clearly measures the impact of the journal as a results of its citation. The 100 percentage is scaled into Quartiles. Q_1_, Q_2_, Q_3_ and Q_4_. Citescore is basically the average number of citations per document that a publication title receives over a three-year period. Journals with high percentiles (Q_1_) are higher desirable because of their high impact. Furthermore other metrics include document-and-citation count and percentage cited.

Generally, Citescore metrics comprise of Citescore, Citescore tracker, Citescore percentile, Citescore Quartiles, Citescore rank, Citation count, document count and percentage cited. It is comprehensive, transparent, current and relevant.

The high values of the skewness in Table 1 imply that the difference among the statistic of the first moment is large. Similarly the high values of the Kurtosis imply the some of the observations are far from the mean.

## 2 Experimental design, materials and methods

The data is openly available at the various webpages of the journals. The data was subsequently extracted and transferred to an Excel file. The stated affiliations posted on the website for the different editorial board members formed the basics for their classifications to their respective continents. In addition, it must be noted that the six continents are differed largely in population, education and development level. The data does not consider the variances because of the following: firstly, the editorial board members are recruited based on their expertise and not based on their country of origin or affiliation. This means that it is possible for all the affiliations of the board members to be the same. This is quite different from paper publication because countries with larger population are most likely to send articles for publication. Secondly, only the official affiliations stated by the editorial board members were obtained which may be different from their country of origin. Lastly, the gender was not considered because it was not officially stated by the publisher.

Again, the Citescore and journal Citescore percentiles were extracted from www.scopus.com.Journals without Citeescore and percentiles were also chosen as long as they are currently abstracted and indexed in Scopus.

The statistical analysis was done to explore the pattern of distribution. Some other statistical analysis can be applied based on the research aim of the researchers. See [1], [2], [3], [4], [5], [6], [7], [8], [9], [10], [11], [12], [13], [14], [15], [16], [17], [18], [19], [20], [21], [22], [23], [24], [25], [26], [27], [28], [29] for details.

### 2.1 Chi-square test of goodness of fit

Chi-square goodness of fit is often used to assess the observed data differs significantly from the expected. It can be used in quality assurance to test the level of compliance to stated policies or standards. The test is used to change in monitoring imbalances in editorial board composition across the continents. The null hypothesis is that there is absence of imbalance in the editorial composition and the alternative hypothesis is the reverse. This is presented in Table 2.

**Table 2:** Chi-square goodness of fit test for the editorial board composition of the Hindawi journals indexed in Scopus. Remarks: In all the continents, there is presence of imbalance in the editorial composition across the continents as seen in the values of the *p*-values. Moreover, the researcher can defined the expected observation based on the policy guiding editorial composition of journals. For example, the editorial board composition of some journals may be based on the quota system. Also the result may be conducted on each of the individual journals.

**Test Statistics**	NAM	EURO	ASIA	SAM	AUST	AFR
Chi-Square	178.133	261.844	340.178	877.733	583.167	621.500
df	33	58	31	11	14	6
Asymp. Sig.	0.000	0.000	0.000	0.000	0.000	0.000

### 2.2 Correlation between the editorial board composition and the Citescore and journal percentile

The Kendall tau and Spearman rank correlation coefficients were used. The Pearson correlation was not used because the data is highly skewed as seen in Table 1 and as such, normality cannot be assumed. Also the hypothesis is based on the p value equals 0.05.

The correlation coefficient for the total editorial board composition and the Citescore is −0.022110 while the *p*-value of 0.776704 while the correlation coefficient for the total editorial board composition and the percentile is 0.095 with the *p*-value of 0.222. These imply that the Citescore and the percentile of the journals are independent of the total editorial board composition.

The correlation coefficient for the editorial board composition (NAM) and the Citescore is 0.041 while the *p*-value of 0.445 while the correlation coefficient for the editorial board composition (NAM) and the percentile is 0.075 with the *p*-value of 0.164. These imply that the Citescore and the percentile of the journals are independent of the editorial board composition (NAM).

The correlation coefficient for the editorial board composition (EURO) and the Citescore is −0.056 while the *p*-value of 0.288 while the correlation coefficient for the editorial board composition (EURO) and the percentile is 0.038 with the p-value of 0.474. These imply that the Citescore and the percentile of the journals are independent of the editorial board composition (EURO).

The correlation coefficient for the editorial board composition (ASIA) and the Citescore is −0.184 while the p-value of 0.001 while the correlation coefficient for the editorial board composition (ASIA) and the percentile is −0.112 with the *p*-value of 0.025. These imply that the Citescore and the percentile of the journals are dependent of the editorial board composition (ASIA).

The correlation coefficient for the editorial board composition (SAM) and the Citescore is −0.102 while the *p*-value of 0.092 while the correlation coefficient for the editorial board composition (SAM) and the percentile is −0.094 with the *p*-value of 0.122. These imply that the Citescore and the percentile of the journals are independent of the editorial board composition (SAM).

The correlation coefficient for the editorial board composition (AUST) and the Citescore is −0.004 while the *p*-value of 0.950 while the correlation coefficient for the editorial board composition (AUST) and the percentile is 0.057 with the *p*-value of 0.331. These imply that the Citescore and the percentile of the journals are independent of the editorial board composition (AUST).

The correlation coefficient for the editorial board composition (AFR) and the Citescore is −0.163 while the *p*-value of 0.008 while the correlation coefficient for the editorial board composition (AFR) and the percentile is −0.087 with the *p*-value of 0.160. These imply that the Citescore of the journals are dependent of the editorial board composition (AFR) and the percentile of the journals is independent of the editorial board composition (AFR).

## References

[1] Burgess T.F., Shaw N.E. (2010). Editorial board membership of management and business journals: a social network analysis study of the Financial Times 40. Br. J. Manag..

[2] Ò. Miró, P. Burbano, C.A. Graham, D.C. Cone, J. Ducharme, A.F.T. Brown, F.J. Martín-Sánchez, Analysis of h-index and other bibliometric markers of productivity and repercussion of a selected sample of worldwide emergency medicine researchers. 10.1136/emermed-2016-205893 27565195

[3] Vošner H.B., Kokol P., Bobek S., Železnik D., Završnik J. (2016). A bibliometric retrospective of the journal computers in human behavior (1991–2015). Comput. Hum. Behav..

[4] Petersen J., Hattke F., Vogel R. (2017). Editorial governance and journal impact: a study of management and business journals. Scientometrics.

[5] Okagbue H.I., Adamu M.O., Oguntunde P.E., Opanuga A.A., Adebiyi A.A., Bishop S.A. (2017). Datasets on the statistical properties of the first 3000 squared positive integers. Data Brief..

[6] Garg K.C., Pali S. (2016). A preliminary investigation of editorial gatekeeping of CSIR-NISCAIR journals. Ann. Libr. Inf. Stud..

[7] Jokić M., Sirotić G. (2016). Do the international editorial board members of croatian social sciences and humanities journals contribute to their visibility?. Medij-. Istraz..

[8] Okagbue H.I., Opanuga A.A., Adamu M.O., Ugwoke P.O., Obasi E.C.M., Eze G.A. (2017). Personal name in Igbo culture: a dataset on randomly selected personal names and their statistical analysis. Data Brief..

[9] Wicherts J.M. (2016). Peer review quality and transparency of the peer-review process in open access and subscription journals. PloS one.

[10] Metz I., Harzing A.W., Zyphur M.J. (2016). Of journal editors and editorial boards: who are the trailblazers in increasing editorial board gender equality?.. Br. J. Manag..

[11] Okagbue H.I., Adamu M.O., Oguntunde P.E., Opanuga A.A., Owoloko E.A., Bishop S.A. (2017). Datasets on the statistical and algebraic properties of primitive Pythagorean triples. Data Brief..

[12] Cummings S., Hoebink P. (2017). Representation of academics from developing countries as authors and editorial board members in scientific journals: does this matter to the field of development studies?. Eur. J. Dev. Res..

[13] Rösing C.K., Junges R., Haas A.N. (2014). Publication rates of editorial board members in oral health journals. Braz. Oral. Res..

[14] Schubert A. (2017). Power positions in cardiology publications. Scientometrics.

[15] Ortega J.L. (2017). Are peer-review activities related to reviewer bibliometric performance? A scientometric analysis of Publons. Scientometrics.

[16] Okagbue H.I., Opanuga A.A., Oguntude P.E., Ugwoke P.O. (2017). Random number datasets generated from statistical analysis of randomly sampled GSM recharge cards. Data Brief..

[17] Schisterman E.F., Swanson C.W., Lu Y.L., Mumford S.L. (2017). The changing face of epidemiology: gender disparities in citations. Epidemiology.

[18] Dhanani A., Jones M.J. (2017). Editorial boards of accounting journals: gender diversity and internationalisation. Account. Audit. Account. J.

[19] Petersen J. (2017). How innovative are editors?: evidence across journals and disciplines. Res. Eval..

[20] Wessa P. (2016). Variability (v1.0.7) in Free Statistics Software (v1.2.1). http://www.wessa.net/rwasp_variability.wasp/.

[21] Pisoschi A.M., Pisoschi C.G. (2016). Is open access the solution to increase the impact of scientific journals?. Scientometrics.

[22] Okagbue H.I., Adamu M.O., Oguntunde P.E., Opanuga A.A., Rastogi M.K. (2017). Exploration of UK Lotto results classified into two periods. Data Brief..

[23] Shideler G.S., Araújo R.J. (2017). Reviewer interest in a manuscript may predict its future citation potential. Scientometrics.

[24] Sarigöl E.E., Garcia D., Scholtes I., Schweitzer F. (2017). Quantifying the effect of editor–author relations on manuscript handling times. Scientometrics.

[25] Forrester J.P., Watson S.S. (1994). An assessment of public administration journals: the perspective of editors and editorial board members. Public Adm. Rev..

[26] Nisonger T. (2002). The relationship between international editorial board composition and citation measures in political science, business, and genetics journals. Scientometrics.

[27] Oguntunde P.E., Okagbue H.I., Adamu P.I., Oguntunde O.A., Oluwatunde S.J., Opanuga A.A. (2018). Statistical analysis of bank deposits dataset. Data Brief.

[28] Park J.Y., Nagy Z. (2018). Data on the interaction between thermal comfort and building control research. Data Brief.

[29] Okagbue H.I., Atayero A.A., Adamu M.O., Opanuga A.A., Oguntunde P.E., Bishop S.A. (2018). Dataset on statistical analysis of editorial board composition of Hindawi journals indexed in Emerging sources citation index. Data Brief.

